# Prognosis by Prophecy—A Memoir of Carl Jung

**Published:** 1990-12

**Authors:** Christopher Davidson


**PROGNOSIS BY PROPHECY**
**A MEMOIR OF CARL JUNG**

Headache, vomiting and papilloedema, these according to Wheeler & Jack's Handbook of Medicine, were the cardinal features of cerebral tumour, but the authors quickly tired of the subject after a page or two, and not knowing what papilloedema was, so did we, clinical clerks in the early thirties fresh from the Medical School with our new stethoscopes and clean white coats. Looking back, this was the first of the triads with which teaching tended to be larded in those days, later we were to meet Charcot's nystagmus, intention, ????? and staccato speech, and the tragic presentation by children in their second year of life of progressive listlessness, vomiting and squint, fortunately never seen nowadays. Later we came to know more about cerebral tumour in the wards, but it was not until I came across a copy of the Post-Graduate Medical Magazine devoted entirely to the pathology of the condition by Dorothy Russell that one realised the essentially sinister nature of the condition, and that only the small proportion of patients lucky enough to have a meningioma had an chance of survival.

Forty years after Wheeler & Jack and perhaps two or three scores of intra-cranial tumours later, there was little more to be said about prognosis, so much so that at the time of my retirement I was able to remember the clinical details of every one of my patients who had survived, all three of them. One was easy, calcification in a parietal meningioma, the second memorable in that I failed to recognise the patient sitting outside the ward in her new wig, all within a month of her first symptom, an epileptic attack. And finally and unforgetable, the first in time way back in the early fifties. A middle-aged man, comfortably off in the Bradford wool trade, he had gradually developed a left hemiparesis and it seemed likely that he had a right-sided cerebral tumour. Our consultant neuro-surgeon agreed and explained to the patient that an exploratory operation was necessary, but only then would it be possible to say whether or not the operation would be successful. Our patient understood the difficulty but asked that he might be allowed a little time to think about it; in the meantime the operation was provisionally arranged for a day or two later. On the afternoon before, when I called to hear his decision, he told me a remarkable story. For a number of years it seemed, his business had been able to look after itself, and he had developed the habit of spending several months each year in contemplative retreat somewhere in southern France where over the years he had made the acquaintance of a number of eminent psychologists, among them Jung, yes the veritable Karl himself. And so, when operation was contemplated, he had been in touch with Jung for his assessment of the problem and advice. The immediate answer for me was that Jung would consider his friend and our patient during the coming night, then if I would ring him at a Geneva number at eight precisely on the following morning he would give me his decision. I duly rang; yes, we must go ahead, of a certainty the operation would be successful. Thus encouraged, my colleague went ahead, the tumour was of course, a meningioma and the prophecy was fulfilled.

CHRISTOPHER DAVIDSON

